# Multiplexed Millimeter Wave Communication with Dual Orbital Angular Momentum (OAM) Mode Antennas

**DOI:** 10.1038/srep10148

**Published:** 2015-05-19

**Authors:** Xiaonan Hui, Shilie Zheng, Yiling Chen, Yiping Hu, Xiaofeng Jin, Hao Chi, Xianmin Zhang

**Affiliations:** 1 Department of Information Science and Electronic Engineering, Zhejiang University, Hangzhou 310027, China

## Abstract

Communications using the orbital angular momentum (OAM) of radio waves have attracted much attention in recent years. In this paper, a novel millimeter-wave dual OAM mode antenna is cleverly designed, using which a 60 GHz wireless communication link with two separate OAM channels is experimentally demonstrated. The main body of the dual OAM antenna is a traveling-wave ring resonator using two feeding ports fed by a 90° hybrid coupler. A parabolic reflector is used to focus the beams. All the antenna components are fabricated by 3D printing technique and the electro-less copper plating surface treatment process. The performances of the antenna, such as S-parameters, near-fields, directivity, and isolation between the two OAM modes are measured. Experimental results show that this antenna can radiate two coaxially propagating OAM modes beams simultaneously. The multiplexing and de-multiplexing are easily realized in the antennas themselves. The two OAM mode channels have good isolation of more than 20 dB, thus ensuring the reliable transmission links at the same time.

In 1992, Allen *et al.* recognized that the light beams with the transverse azimuthal dependence of exp(-*jlφ*) carry orbital angular momentum (OAM)^1^. Since then, the applications using electromagnetic beams carrying OAM are widespread^2^,^3^,^4^,^5^,^6^,^7^,^8^,^9^,^10^,^11^,^12^,^13^. The early studies concerning the interaction of OAM with matter evoked the applications of optical tweezers^6^, optical drive of micro-machines^7^, atoms trapping and guiding^8^. Besides the noticeable potential in communications, the recent advances for OAM carrying beams refer to rotational Doppler shift effect^9^, which provides a new way to detect rotation speed, the remarkable stimulated emission depletion (STED) microscopy^10^,^11^, where the OAM beams perform as depletion mask spot to improve the microscopy resolution, and the field of quantum physics^12^,^13^.

The fundamental idea for OAM to be used in communications lies in that it can have unbounded eigenstates, thus is allowed in principle to offer many channels so as to increase the transmission capacity. Comparing with spin angular momentum (SAM), which has only two orthogonal states, applying OAM to improve the communication capacity brings a bright prospect to both academic and industrial community^14^,^15^. Great progress has been made in optical regime recently^16^,^17^. Different OAM beams have been multiplexed in free space optical communication or specially designed fiber communication to achieve high spectral efficiency and capacity of Tbits^-1^ 18,19. However, it is only recently that OAM found potential use in the low frequency radio domain^20^,^21^,^22^,^23^. Though the basic physical properties of the EM fields can be translated from optics to radio, differences do exist between the two frequency regimes.

The multiplexing and de-multiplexing of the OAM radio waves are the big bottlenecks for the OAM based wireless communication. The OAM beams can be generated easily in optical regime, especially when the programmable spatial light modulator (SLM) is applied. The existing optical devices can also provide mature schemes to manipulate the OAM beams such as combining, splitting, collimation, concentrating, and detecting^17^,^24^,^25^,^26^. Hence the OAM optical beams can be deftly multiplexed and de-multiplexed. However, when it comes to the radio frequency regime, as the wavelength is much longer than the optics, it is difficult to manipulate the OAM radio beams, such as beam combining and splitting, so that the coaxially transmitting cannot be easily ensured. The specially designed devices are needed and may bring great insertion loss in the link, which will reduce the efficiency.

Several methods have been reported to generate microwave or millimeter-wave beams carrying OAM. The spiral phase plate (SPP) is a widely used scheme because of its simple structure^27^,^28^,^29^,^30^. Its design idea is the same with that in optical regime, using which the phase of the transmitting wave increases in proportion to the azimuthal angle *φ* around the center^31^,^32^,^33^,^34^. However, multiplexing and de-multiplexing of the OAM-carried radio waves are not easy to be implemented when multiple SPPs are used. Furthermore, the wide-angle directivity of the beam transmitted through SPP is inappropriate for long distance propagation, especially in low frequency region^35^,^36^,^37^,^38^. The OAM wireless communication experiment based on the spiral parabolic antenna was demonstrated at microwave band^23^. The antenna was transformed by a commercial parabolic antenna which is easily to be manufactured. In fact, the main idea of this method is similar to that of the SPP, transmitting the different OAM beams within a same aperture is still a problem. The circular antenna array is another way to generate OAM beams^39^,^40^,^41^, it can radiate different OAM beams simultaneously. However, the exact controlled phase shift feeding network is necessary, which will increase both the cost and the complexity of the system in practice use.

The OAM based radio communication is a new territory, which needs deeply exploration. A new method to generate the OAM radio wave, which can also provide an easy scheme to realize the multiplexing and de-multiplexing is of great significance for OAM based wireless communication.

In this paper, the OAM multiplexed communication links are demonstrated based on a novel millimeter-wave dual OAM mode antenna. The main body of this dual OAM mode antenna is a traveling-wave slot antenna based on ring cavity resonator. Using two feed ports fed by a 2 × 2 waveguide 90° hybrid coupler, dual OAM modes of millimeter-wave can be generated and transmitted coaxially. A ring parabolic reflector is employed to focus the OAM beams. The design principle, design procedure and the manufacture process are firstly elucidated; the characteristics of the antenna, such as the near field radiation, the far field directivity, the S parameter of the two ports are then measured. Based on this dual OAM mode antenna, 60 GHz communication experiments with one channel of high definition (HD) video and the other of square wave modulated wave are finally performed.

## Results

### Structure of the dual OAM modes antenna

A circular traveling-wave loop antenna with its phase change along the circle of *lφ* was demonstrated to generate the OAM carried beams recently^42^. To enhance the radiation efficiency, a traveling-wave circular slot antenna based on ring resonant cavity is adopted. The ring resonant cavity is a metallic ring cavity manufactured by twisting a section of rectangular waveguide with wide wall *a* and narrow wall *b*. The waveguide is operated in the fundamental mode, TE_10_. To generate ±*l* modes OAM beams, approximately, the circumference *C* of the ring resonator can be calculated by


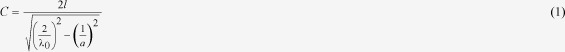


where *λ*_0_ is the wavelength in vacuum.

**Figure 1:**
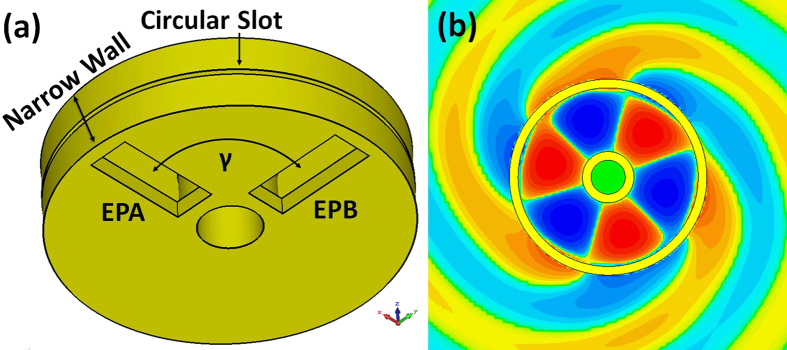
The ring resonator. (**a**) The waveguide ring resonator with two feeding ports (EPA and EPB). The angle between two feeding ports is γ. The circular slot is in the narrow wall of the resonator. (**b**) The traveling-wave electric field distribution in the resonator and the radiation field from the slot.

In order to excite a travelling wave in the ring resonator, two feeding ports with the same signal amplitude but of 90° phase difference are used, named EPA and EPB in Fig. 1a. The angle between the two feeding ports is γ,


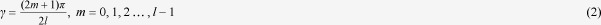


With such feeding, a clockwise or anti-clockwise traveling wave distributed fields can be excited in the ring resonator depending on the phase difference of +90° or −90°. When a circular slot is cut on the narrow wall of the waveguide, the electromagnetic field will radiate from the slot, which constitutes a traveling wave circular slot antenna for OAM wave generation, as shown in Fig. 1b. The radiated wave will carry +*l* or −*l* OAM mode according to the clockwise or anti-clockwise traveling wave in the resonator.

**Figure 2:**
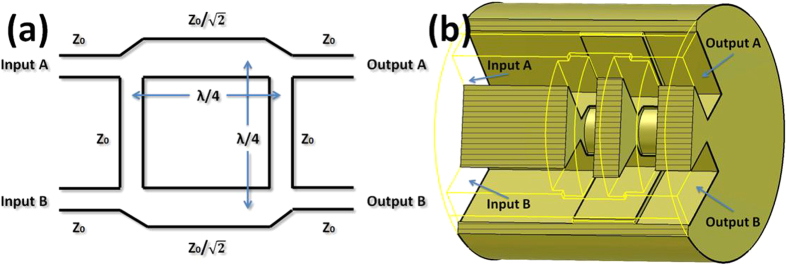
The 90° hybrid coupler is designed as the feeding network. (**a**) The schematic of the 90° hybrid coupler with two input ports and two output ports. (**b**) The waveguide 90° hybrid coupler model to match the structure of the ring resonator shown in the Fig. 1a.

A 2 × 2 waveguide 90° hybrid coupler is used as the feeding network. Figure 2a shows the principle schematic. When all ports are matched, the signals from the two output ports A and B will have the same magnitude but of +90° or −90° phase difference, which is decided by the input port A or B. If the input port A is fed, the phase difference between the signals from output ports A and B is +90°, while the input port B is fed, the phase difference is −90°. To match the structure of the ring resonator shown in Fig. 1, the waveguide 90° hybrid coupler is custom designed in a cylindrical body, as shown in Fig. 2b. The impedance is adjusted by regulating the dimension of narrow wall. The output port A and B of the 2 × 2 waveguide 90° hybrid coupler are connected with EPA and EPB of the ring resonator. The feeding option of the input port A or B for the waveguide 90° hybrid coupler decides the radiated OAM mode of +*l* or −*l*. Much more interestingly, when the two input ports A and B are excited simultaneously, the dual OAM modes of ±*l* beams will radiate at the same time. A dual OMA mode antenna is obtained without extra multiplexing.

**Figure 3:**
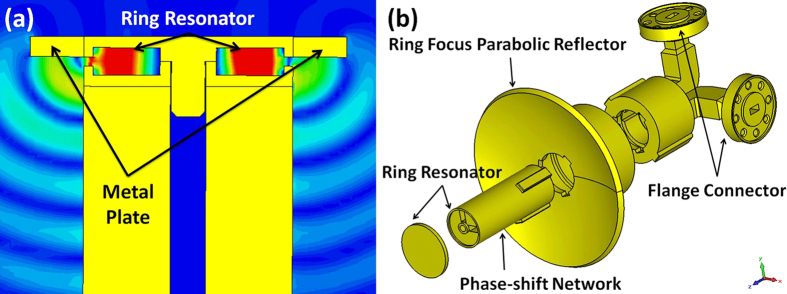
(**a**) The section view of the slot-loaded resonator and its radiation field. (**b**) The explosion view of the antenna. It is composed of four parts, including the ring resonator and metal plate, the phase-shift network (90° hybrid coupler), the ring focus parabolic reflector, and the flange connectors.

Figure 3a shows the section view of the slot-loaded resonator and its electromagnetic field radiation. A metal plate is designed to guide the radiation field to a ring focus parabolic reflector for concentrating OAM beams into a favorable directivity. The explosion view of the whole dual OAM mode antenna is shown in Fig. 3b. It is composed of four parts, including the ring resonator and metal plate, the phase-shift network (90° hybrid coupler), the ring focus parabolic reflector, and the flange connectors.

**Figure 4:**
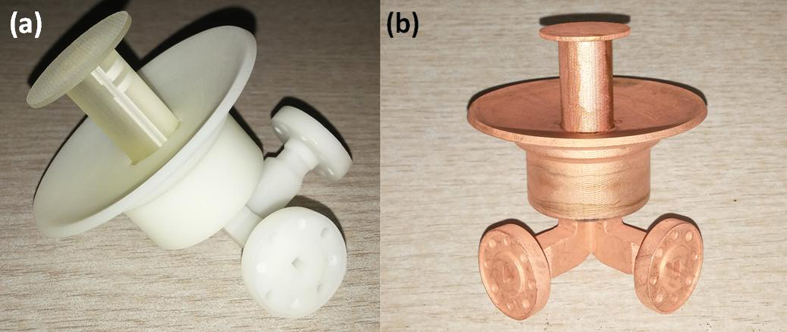
The fabricated dual OAM states antenna (l = ±3). (**a**) 3D printed (photosensitive polymer) antenna blank without metallization. (**b**) after the electro-less copper plating surface treatment process. The diameter of the ring focus parabolic reflector is ~50 mm.

The antenna is designed to generate 60 GHz millimeter wave carrying dual OAM states of *l* = ±3. The simulation work is completed by using CST Microwave Studio. After that this antenna is fabricated by 3D printing technique, as shown in Fig. 4a. The printing resolution is 0.016 mm which guarantees the precision of the antenna working in 60 GHz band. The polymer surface is then metalized by the electro-less copper plating surface treatment process. The thickness of the copper film is about 5 μm which is far greater than the skin depth. The accomplished dual orbital angular momentum mode antenna is shown in Fig. 4b.

### Measurement of the antenna

**Figure 5:**
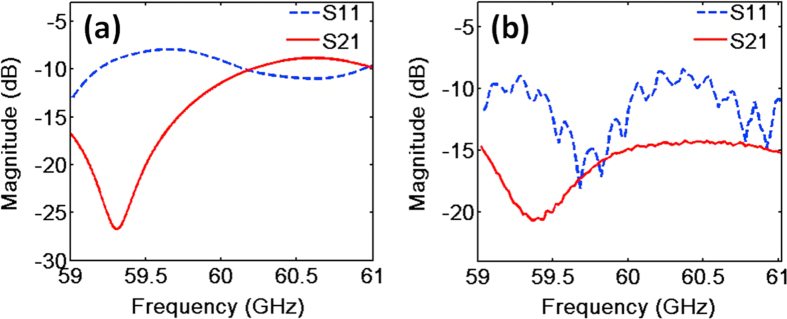
The S-parameters of the antenna, S11 is the return loss and S21 represents the crosstalk between two ports of the dual OAM antenna. (**a**) The simulation results. (**b**) The measurement results.

The S-parameters for the two ports of the dual OAM mode antenna of *l* = ±3 are firstly measured. The measurement is implemented by a vector network analyzer (VNA, R&S ZVA 67). Figures 5a,b show the simulation and measurement results, respectively. S_11_ is the return loss. It is relatively low within the designed frequency band, which means the antenna has a low reflectivity. S_21_ represents the crosstalk between two ports. The lower S_21_ ensures higher isolation of the two OAM channels at the transmission end. The difference between the simulation prediction and the actual measurement is mainly due to the assemble precision and the surface resistance of copper film. Although the antenna remains the low reflection in a wide band, the ring resonator and the 90° hybrid coupler are narrow devices which will restrict the bandwidth of the antenna. The working frequency deviation will result in the mismatch of the 90° hybrid coupler and the ring resonator, and deteriorate the performances of the antenna.

**Figure 6:**
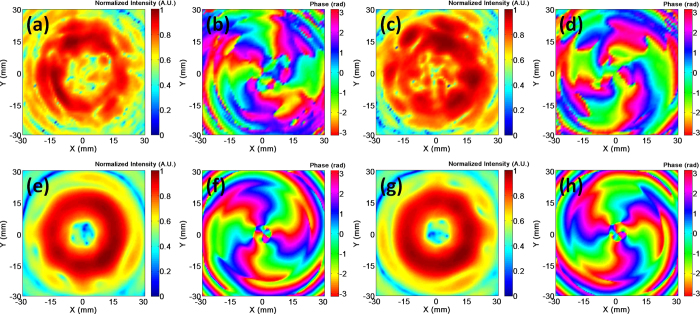
The near-field radiation for OAM states of *l* = ±3. (**a**)~(**d**) are the measured results; (**e**)~(**h**) are the simulation results. (**a**), (**c**), (**e**) and (**g**) are the amplitudes of the radial component of the electric field, and (**b**), (**d**), (**f**) and (**h**) are the phase of the radial component of the electric field.

The near-field radiation patterns of the manufactured antenna are measured by VNA. An open end waveguide is used as the probe that is set on a 3D platform (See Supplemental Fig. 1). Figures 6a–6d present the measured near-field pattern for OAM states of *l* = ±3. For comparison, the near field patterns of the antenna are also simulated in CST Microwave Studio, as shown in Figs. 6e–6h. Figures 6a,c,e,g are the amplitudes while Figs. 6b,d,f,h are the phases of the radial components of the electric fields. They are obtained from the plane (60 mm × 60 mm) at 20 mm far away from the antenna. The operating frequency is 60 GHz. It is well known that an OAM beam exhibits the amplitude with a donut shaped contour, and the phase with vortex trajectories and a singularity at center. Figure 6 shows that the measured results are in good agreements with the simulated ones. Both results verify that the radiation of the antenna carry OAM. Moreover, the phase distributions demonstrate that the OAM mode is +3 or −3. The animated radiation from the dual OAM mode antenna can be seen in Supplementary video 1 and Supplementary Note 1.

**Figure 7:**
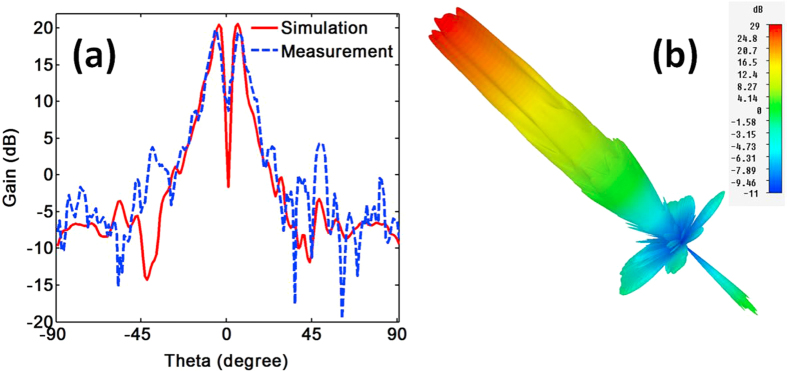
(**a**) The measurement result (blue dash line) and the simulation result (red solid line) for the far-field directivity diagram of the dual OAM antennas with the parabolic reflector diameter of 100 mm. The measured direction angle, gain and 3 dB angular width of the main lobe are ~6°, 20 dB, and ~4.5°, respectively. (**b**) The simulated 3D pattern for the antenna with reflector diameter of 200 mm.

Since the electromagnetic field is very weak near the center of OAM beam due to the phase singularity, a smaller main lobe direction angle is desired for wireless transmission. With the help of the ring focus parabolic reflector, the OAM beams can be effectively concentrated. Figure 7a shows the measured (blue dash line) and the simulated (red solid line) far-field directivity diagram for the dual OAM antenna with the parabolic reflector diameter of 100 mm. It can be seen that the measurement fits well with the simulation, especially the main lobe. The measured direction angle, gain and 3 dB angular width of the main lobe are ~6°, 20 dB, and ~4.5°, respectively. The relation between the reflector diameter *D* and the far field directivity is thoroughly studied (See Supplementary Fig. 2 and Supplementary Table 1). The larger the reflector diameter is, the better the performances. When the reflector diameter is 200 mm, the gain of the antenna is 27.4 dB and the divergence angle is only 2.8°. Figure 7b shows the simulated 3D pattern for the antenna with reflector diameter of 200 mm.

### Communication Experiment

**Figure 8:**
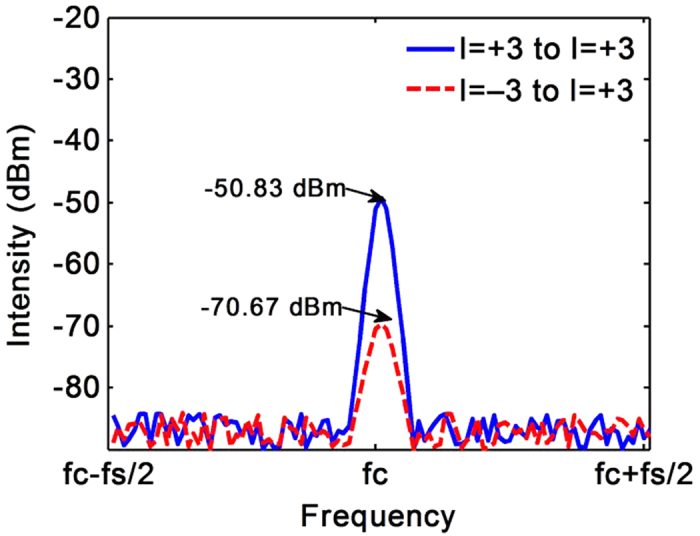
The output signals from the l = +3 port of the receiving antenna measured by a spectrum analyzer. The blue solid line is the receiving signal intensity when the signal is launched into the l = +3 port of the transmitting antenna, and the red dash line is that when the signal launched into the l = –3 port. fc = 60 GHz, fs = 20 kHz.

The wireless transmission experiments are performed based on a pair of dual OAM mode antennas. Firstly, the isolation of the two OAM channels is tested. A dual OAM mode antenna with the reflector diameter of 100 mm acts as the transmitting antenna, and the other one with the reflector diameter of 200 mm acts as the receiving antenna. The two antennas are concentrically placed with 4 m apart. The excitation signal is generated by Agilent E8257D with frequency of 60 GHz and the power of 0 dBm. The output signals from the *l* = +3 port of the receiving antenna are measured by a spectrum analyzer (Agilent 8563EC and 11974V) and shown in Fig. 8. The blue solid line is the receiving signal intensity when the 60 GHz signal is launched into the *l* = +3 port of the transmitting antenna, the red dash line is that when the signal is launched into the *l* = –3 port. It can be seen that the isolation is about 20 dB. If the distance between two antennas is shortened, the isolation will be better. (The photos of the output signal taken from the spectrum analyzer screen are shown in the Supplementary Fig. 3.)

**Figure 9:**
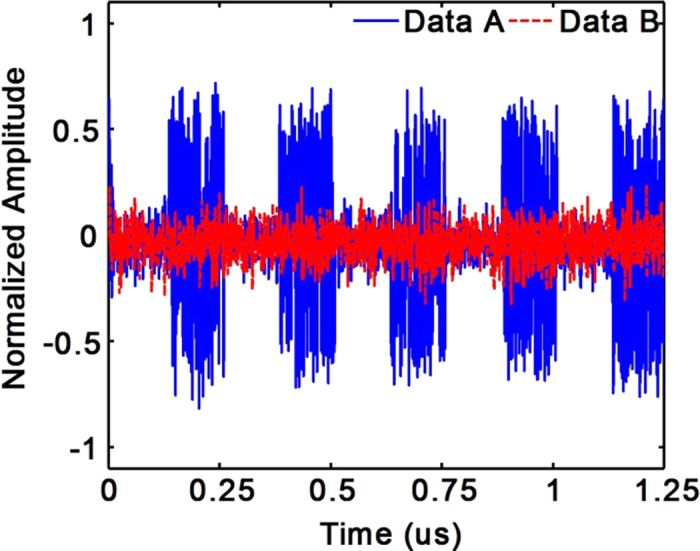
The signals are monitored by a digital communication analyzer. The data A (blue solid line) are the signals when the modulated signal is launched into to the *l* = +3 port of the transmitting antenna, the data B (red dash line) are the signals A when the modulated signal is launched into the *l* = –3 port of the transmitting antenna.

A 4 MHz square wave modulated transmission through this 60 GHz wireless OAM channel is then investigated. The signals are monitored by a digital communication analyzer (HP 83480A). Data A (blue solid line) in Fig. 9 is recorded when the modulated signal is launched into the *l* = +3 port of the transmitting antenna, and data B (red dash line) is that when the modulated signal is launched into the *l* = –3 port. It is seen that data A shows a clear square wave with the period of 0.25 μs, which means that a successful transmission is established. The flatness of Data B proves the low cross talk and ensures the transmission reliability for the dual OAM channels.

**Figure 10:**
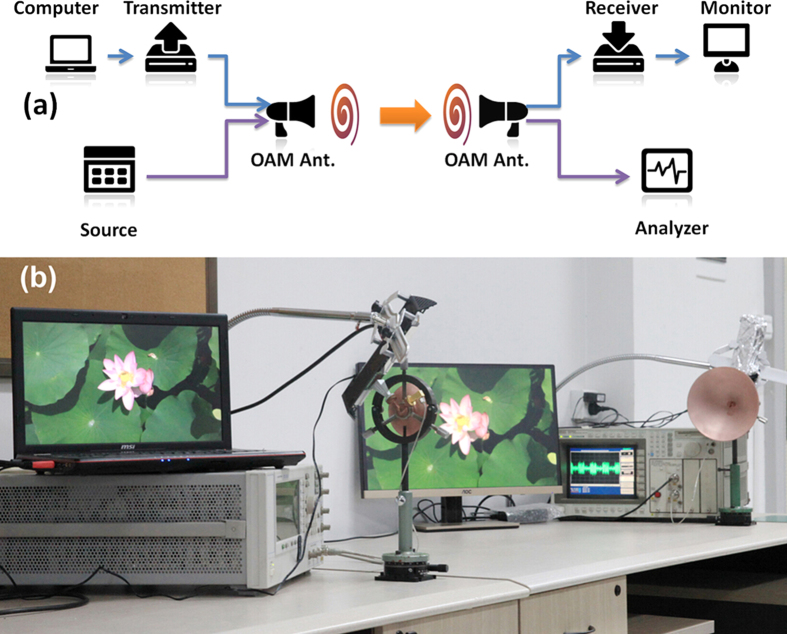
(**a**) The experiment setup of the dual OAM communication link. The *l* = +3 channel carries the square wave modulated millimeter-wave signal, and the *l* = −3 channel carries the HD video signal. (**b**) The experimental photo to demonstrate the successful transmission of two independent channels. The picture on the screen is extracted from the video, Copyright The Admission Office of Zhejiang University.

Figure 10a shows the experimental setup for the two channels OAM wireless communication transmission. The *l* = –3 channel is used to transmit HD video. The video signal output from a computer *via* HDMI interface is modulated on the 60.25-62 GHz band according to Wireless HD protocol. The *l* = +3 channel is used to transmit the 4 MHz square wave modulated 61 GHz millimeter-wave signal. The dual OAM modes carrying different signals are transmitted together and received by the other dual OAM mode antenna at their corresponding ports. The 4 MHz square wave signal is monitored by the digital communication analyzer, and the video signal is demodulated by the Wireless HD receiver and output to a display through a HDMI cable. The distance between the two antennas is 1.4 m. Figure 10b shows the experimental scene. A video is recorded for the detailed experiments (See Supplementary video 2 and Supplementary Note 2). During the experiment, the HD video plays smoothly and the 4 MHz square wave signal is also transmitted clearly at the same time. The bit error rate (BER) of the HD video transmission is investigated by comparing the original data of the HD video with the one after transmitted through the OAM channel. The BER for the 30 frames of HD videos is shown in the Supplementary Fig. 4. It can be seen that the BER is about the level of 10^–4^. The above results show that two separate wireless channels are established successfully based on the dual OAM mode antennas.

## Discussion

A novel millimeter-wave dual OAM mode antenna is demonstrated in this paper. Compared with the already reported method to generate the OAM beam of millimeter-wave, the designed antenna can provide two coaxially propagating OAM modes simultaneously. These two OAM mode channels have good isolation of more than 20 dB, thus provide the reliable transmission links at the same time. In our experiments, the HD video signals and the square wave modulated millimeter-wave signals are transmitted successfully in the corresponding channels. The transmit distance is 1.4 m at the millimeter-wave band of about 60 GHz. Though it is not long enough, we are confident that the performance can be better when all the conditions are optimized. The main reason for the not long transmission distance is that the designed bandwidth is not so good match for the HD video’s frequency.

With the explosively growth of wireless applications, it is of great importance to find good multiplexing scheme for the OAM carried radio waves. The previously reported works on millimeter-wave OAM multiplexing and de-multiplexing were based on the quasi-optical systems^35^,^43^, which may not operate properly with the compact size at the lower radio frequency. In our system, the antenna is proposed based on the antenna theory, and it is able to be easily designed and fabricated at both the microwave and millimeter-wave band. Much more important, the multiplexing and de-multiplexing of dual OAM modes are accomplished in the antenna itself.

Comparing with the traditional polarization multiplexing scheme, the current system may have no extra advantages as only two OAM sates are exploited in the communication. However, based on the proposed structure, multiple slot-loaded ring cavity resonators can be stacked concentrically. With a carefully designed parabolic reflector, more than 2 OAM modes multiplexing and de-multiplexing can be conveniently achieved. In addition, the current communication distance of 1.4 meter is not long enough for the real application. Great efforts should be paid at these works later.

In conclusion, a new idea on the generation and multiplexing of OAM radio waves is proposed and successfully realized. It will have a positive effect on the application potential of OAM wireless communication.

## Methods

The OAM beams are emitted by the dual OAM antenna, which is designed by CST Microwave Studio, and fabricated by photosensitive polymer 3D printing technique and the electro-less copper plating surface treatment process.

The S-parameters are measured by the R&S ZVA-67 vector network analyzer and the near-fields are scanned by the open end waveguide antenna which is set on a 3D platform. The far-field directivity diagrams are measured by the standard waveguide horn antenna and the spectrum analyzer (Agilent 8563EC and 11974V). The communication experiments are based on the Wireless HD equipment and the analog signal generator (Agilent E8257D). The signals are monitored by the digital communication analyzer (HP 83480A).

## Author Contributions

X.Z. and S.Z. conceived the original idea. S.Z. completed the theoretical analysis. X.H. and S.Z. designed the antennas and experiments. X.Z., X.J. and H.C. supervised the experiments. X.H., Y.C. and Y.H. carried out the experiments. X.H, S.Z. and X.Z. produced the manuscript and interpreted the results. All authors participated in discussions and reviewed the manuscript.

## Additional Information

**How to cite this article**: Hui, X. *et al*. Multiplexed Millimeter Wave Communication with Dual Orbital Angular Momentum (OAM) Mode Antennas. *Sci. Rep.* **5**, 10148; doi: 10.1038/srep10148 (2015).

## Supplementary Material

Supplementary Materials

Supplementary Video 1

Supplementary Video 2
