# Supplementary material for: Multiplexed Millimeter Wave Communication with Dual Orbital Angular Momentum (OAM) Mode Antennas
Source: Sci Rep. 2015 May 19;5:10148. doi: 10.1038/srep10148 (PMC4437312; doi:10.1038/srep10148)
Supplement: Supplementary Materials [file srep10148-s1.doc]

Title:

Multiplexed Millimeter Wave Communication with Dual Orbital Angular Momentum (OAM) Mode Antennas

Xiaonan Hui Shilie Zheng* Yiling Chen Yiping Hu

Xiaofeng Jin Hao Chi Xianmin Zhang*

Department of Information Science and Electronic Engineering,

Zhejiang University, Hangzhou 310027, China.

Email: [zhengsl@zju.edu.cn](mailto:zhengsl@zju.edu.cn);

[zhangxm@zju.edu.cn](mailto:zhangxm@zju.edu.cn)

**Supplementary Figures**

**Supplementary Figure 1**


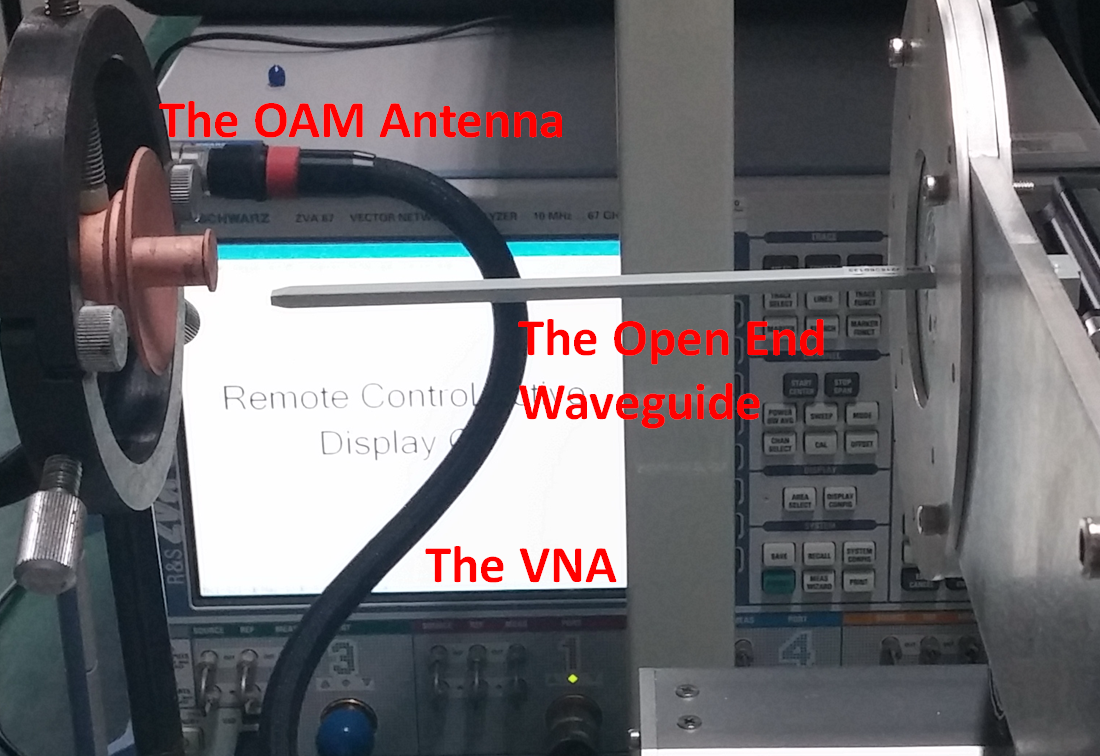


**Supplementary Figure 1** The measurement of the near-fields patterns of the dual OAM antenna It is measured by the vector network analyzer (VNA) and the open end waveguide mounting on a 3D platform. The near-fields are obtained from the plane (60 mm × 60 mm) 20 mm far away from the antenna.

**Supplementary Figure 2**


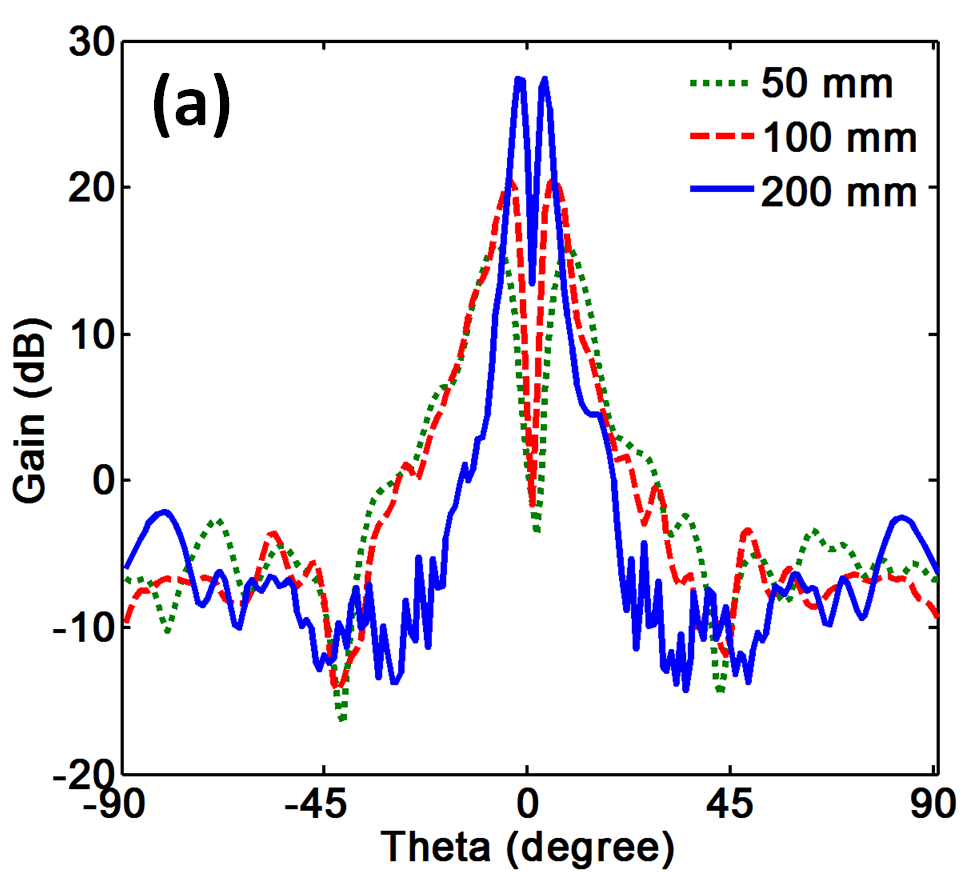


**Supplementary Figure 2** The far-field directivity diagrams of the dual OAM antennas related to different reflector diameters *D*. Green dot line for *D*=50 mm, red dash line for *D*=100 mm, and blue solid line for *D*=200mm. The detailed performances are listed in supplementary Table 1.

**Supplementary Table 1**

Performance of radiation main lobe related to different reflector diameters

| *Reflector Diameter D (mm)* | *Direction angle* | *Gain (dB)* | *3 dB Angular Width* |
| --- | --- | --- | --- |
| 50 | ~7° | 16.2 | ~7.5° |
| 100 | ~5° | 21 | ~4.7° |
| 200 | ~2.8° | 27.4 | ~2.6° |

**Supplementary Figure 3**


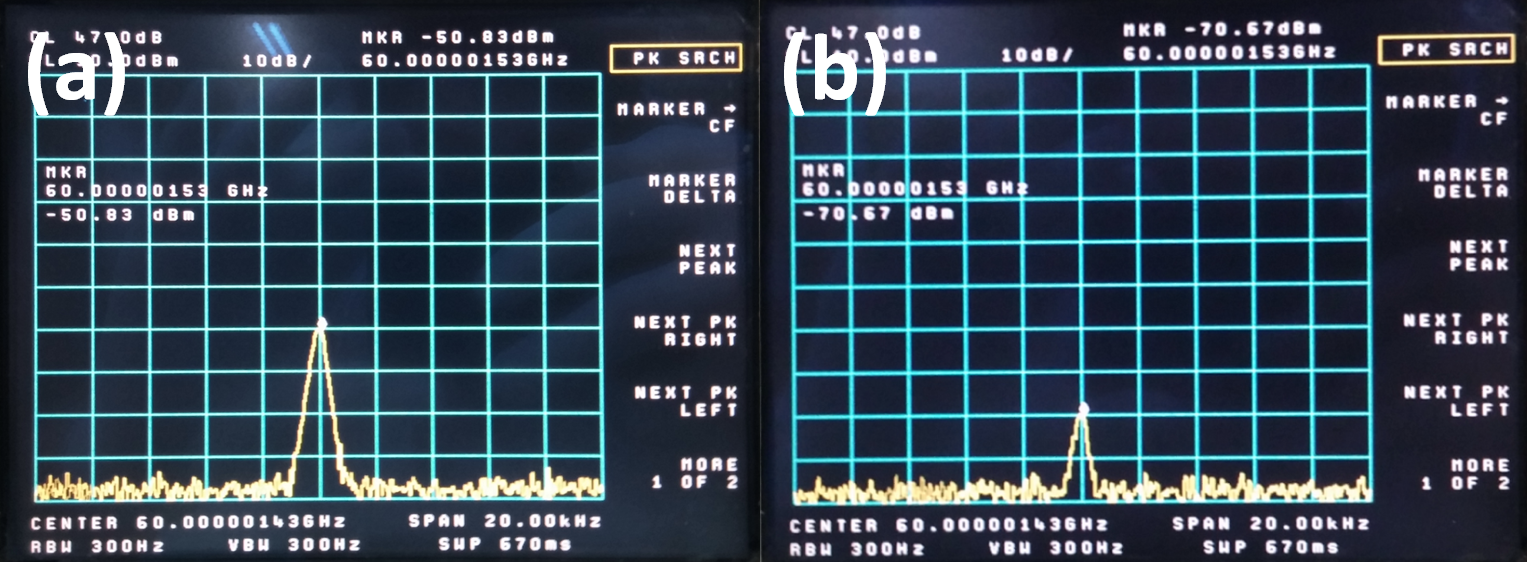


**Supplementary Figure 3** The photos of the output signals intensity from the *l*=+3 port of the receiving antenna measured by a spectrum analyzer. (a) the receiving signal intensity at the receiving *l*=+3 port when the signal is launched into the transmitting *l*=+3 port; (b) the receiving signal intensity at the receiving *l*=+3 port when the signal launched into the transmitting *l*=–3 port. Center frequency=60 GHz, Span=20 kHz.

**Supplementary Figure 4**


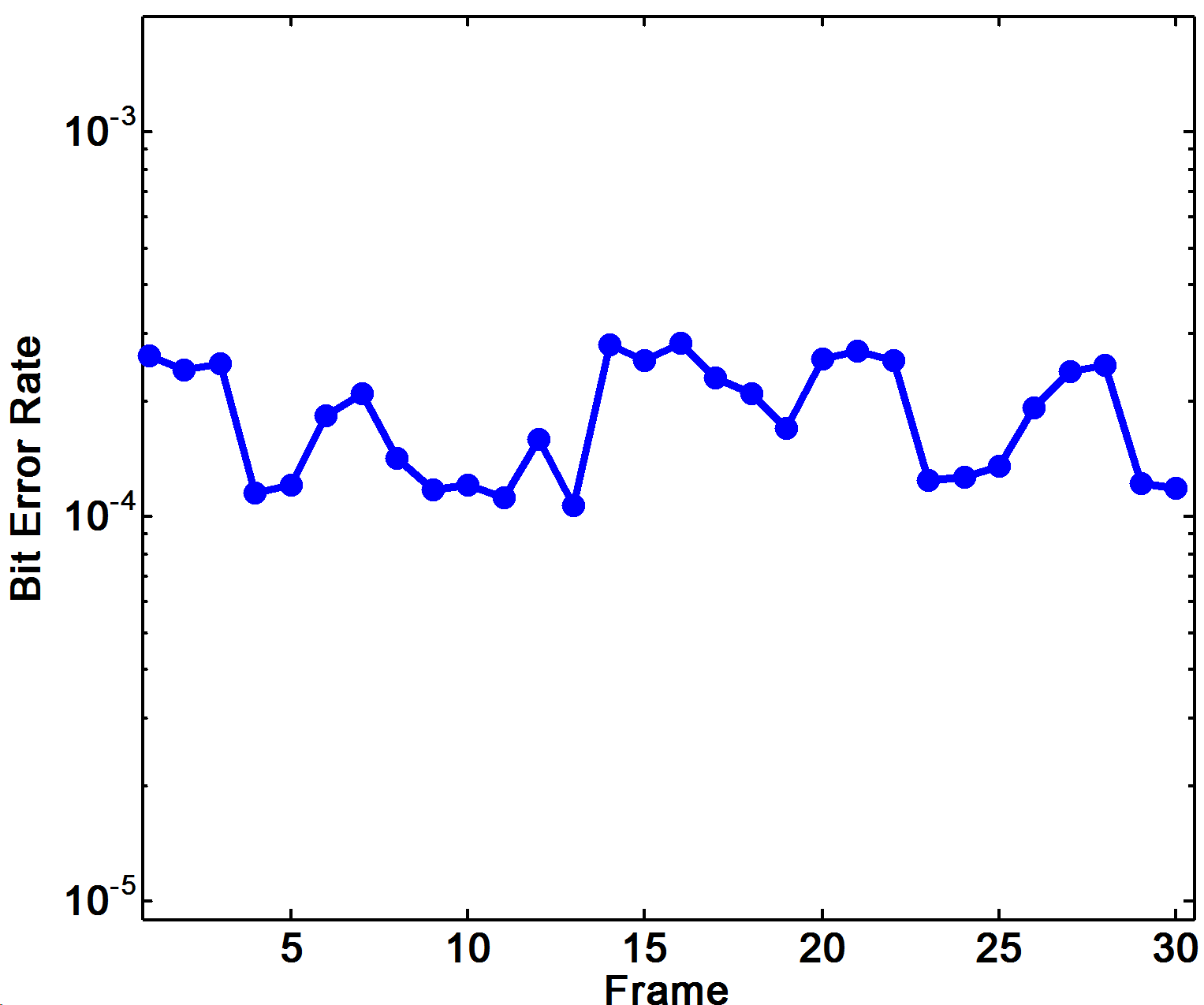


**Supplementary Figure 4** The HD video’s bit error rate (BER) when the multiplex channels are performing. There are 30 frames’ data recorded, and the BER is about the level of 10^-4^.

**Supplementary Video 1**

The animated radiation from the dual OAM mode antenna, uploaded as file “supplementary video1.gif ”

**Supplementary Video 2**

The experimental video demonstrating the simultaneous transmission of the two independent channels, uploaded as file “supplementary video2.mov ”

The HD video signal transmitted as one of the OAM channels is extracted from the video about introduction of Zhejiang University for undergraduate enrollment with permission from the Admission Office of Zhejiang University.

**Supplementary Notes**

**Supplementary Note 1: The animated radiation of the dual OAM mode antenna**

We use the CST Microwave Studio software to demonstrate the dynamic near-field radiation of the dual mode antenna. When the right port of the antenna is excited, the anti-clockwise rotation traveling wave exists in the cavity, and the radiation carries OAM mode of *l*=+3. When the upper port is excited, the clockwise rotation appears, and the radiation carries OAM mode of *l*=-3 (please See in Supplementary video 1).

**Supplementary Note 2: The experiment of multiplexed 60GHz millimeter wave communication based on dual OAM Mode Antennas**

The experiment is carried out by two dual OAM mode antennas. One antenna with the reflector diameter of 100 mm (the smaller one) acts as the transmitting antenna, and the other one with the reflector diameter of 200 mm (the bigger one) acts as the receiving antenna. The two ports of the transmitting antenna are connected to the signal source and the HD video transmitter, respectively. The receiving antenna is connected to the digital communication analyzer and a wireless HD receiver, whose output is displayed on the screen. The distance between the two antennas is 1.4 meter. During the experiment, the HD video plays smoothly and the modulated square carrier is shown on the digital communication analyzer. When the receiving antenna is blocked by a metal plate, the two channel signals are interrupted. And the communication channels reconstruct when the plate is removed.

The HD video signal transmitted is extracted from the video about introduction of Zhejiang University for undergraduate enrollment, with permission from the Admission Office of Zhejiang University.
