# Supplementary figures and images for: Multiplexed Millimeter Wave Communication with Dual Orbital Angular Momentum (OAM) Mode Antennas
Source: Sci Rep. 2015 May 19;5:10148. doi: 10.1038/srep10148 (PMC4437312; doi:10.1038/srep10148)

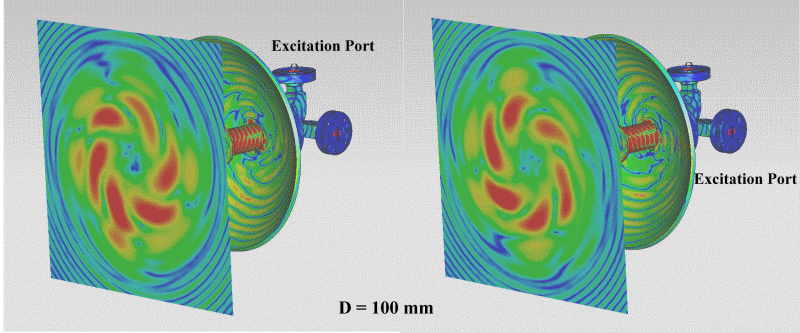

Supplement: Supplementary Video 1 [file srep10148-s2.gif]
